# Exceptional and Durable Responses to TDM-1 After Trastuzumab Failure for Breast Cancer Skin Metastases: Potential Implications of an Immunological Sanctuary

**DOI:** 10.3389/fonc.2018.00581

**Published:** 2018-12-03

**Authors:** Tommaso Giarratano, Federica Miglietta, Carlo A. Giorgi, Vassilena Tsvetkova, Silvia Michieletto, Laura Evangelista, Ilaria Polico, Maria V. Dieci, Valentina Guarneri

**Affiliations:** ^1^Division of Medical Oncology 2, Istituto Oncologico Veneto IRCCS, Padua, Italy; ^2^Department of Surgery, Oncology and Gastroenterology, University of Padua, Padua, Italy; ^3^Anatomy and Histology Unit, Azienda Ospedaliera di Padua, Padua, Italy; ^4^Breast Surgery Unit, Istituto Oncologico Veneto IRCCS, Padua, Italy; ^5^Nuclear Medicine and Molecular Imaging Unit, Istituto Oncologico Veneto IRCCS, Padua, Italy; ^6^Breast Imaging Unit, Istituto Oncologico Veneto IRCCS, Padua, Italy

**Keywords:** metastatic breast cancer, skin metastasis, T-DM1, adotrastuzumab, emtansine, immune microenvironment

## Abstract

Breast Cancer (BC) skin metastases represent a challenging clinical scenario. Although they usually arise when other distant metastases are already present, they may also represent a form of locoregional recurrence (LRR). Systemic therapy in this setting may have a role both in case a radical locoregional approach is unfeasible in order to achieve disease control, and as adjuvant strategy after radical removal of cutaneous lesions, in order to prevent or delay subsequent disease spread. Systemic therapy for HER2+ metastatic BC (MBC) currently relies on anti-HER2 targeted agents. In this context TDM1 is an option in trastuzumab-resistant patients.Here we present 2 cases of isolated skin metastases in patients with HER2+ BC progressing during or early after trastuzumab-based therapy, showing impressive responses to TDM1. We hypothesize that the unique properties of skin immune microenvironment may explain the failure of trastuzumab, which exerts its action also through immunological mechanisms, and the subsequent outlier responses to TDM1, that relies on a partially different mechanism of action.

## Introduction

Although skin metastases arising from non-skin solid malignancies are relatively uncommon (1, 2), they are considerably more frequent among breast cancer (BC) patients compared to other solid tumors, with an overall incidence of ~24% in the largest reported series (2). Skin relapse usually represents a late stage in the course of BC natural history, occurring in most cases when other distant metastases are already present, and it is associated with poor survival rates (2). In this context, the use of systemic therapeutic approach plays a major role. Indeed, the administration of either chemotherapy (CT), endocrine therapy (ET), targeted agents or combinations of them, is aimed at keeping systemic disease under control, especially in case of simultaneous visceral involvement.

However, cutaneous metastases may also appear as a form of isolated locoregional recurrence (LRR) (3–5). When surgical approach is not feasible, palliative systemic treatment is aimed at delaying disease dissemination to other organs/sites and local complications and can be integrated with palliative radiotherapy. However, sometimes, when a radical excision of cutaneous metastases is feasible upfront, it may be considered as the first choice. In this context, systemic therapy may play a role as “adjuvant” treatment after skin metastasis excision. Evidence from the randomized CALOR study showed that “adjuvant” CT after complete excision of BC LRR prolonged both DFS and OS especially in case of HR- BC (6).

In this paper we focused on cases of skin metastases occurring in HER2+ BC patients. Even though HER2-positive (HER2+) metastatic breast cancer (MBC) still represents an incurable disease, the introduction of anti-HER2 targeted agents has dramatically altered its natural history, with increasing improvements in survival rates (7–9).

In the pre-trastuzumab era, HER2 positivity represented an independent predictor for LRR (5, 10). However, after the introduction of anti-HER2 targeted therapy, incidence of LRR in HER2+ patients has dramatically decreased. Indeed, it has recently been reported that the 5y-cumulative incidence of chest wall recurrence as first site of recurrence after mastectomy (with or without synchronous distant metastases) is lower among HER2+ (11, 12) compared to HER2 negative BC patients. In addition, it has been reported that skin represents the first distant site of relapse in ~8% of HER2+ BC patients (13).

Evidence from several randomized clinical trials strongly suggest that there is a survival benefit from continuing some sort of HER2 blockade for metastatic disease in case of progression during or after anti-HER2 targeted therapies (14–17).

Ado-trastuzumab emtasine (TDM-1) is an anti-HER2 targeted antibody conjugate, currently indicated for the treatment of HER2+ MBC patients that have either received trastuzumab for metastatic disease or developped disease recurrence during or within 6 months from the completion of adjuvant trastuzumab-based therapy (18). This molecule consists of trastuzumab plus the cytotoxic DM1 (emtasine), the first selectively and specifically delivering the second inside the HER2+ cancer cells through receptor-mediated endocytosis. Once released inside the tumor cells, DM1 exerts its cytotoxic effect through several mechanisms, such as mitotic arrest, apoptosis, mitotic catastrophe, and disruption of intracellular trafficking, ultimately causing cell death (19).

Herein we describe 2 cases of patients with HER2+ cutaneous disease showing progression during or after trastuzumab-containing regimens and subsequent exceptional responses to TDM1.

## Case presentation

### Patient 1

A 61 years old post-menopausal woman was admitted to our hospital for a locally advanced tumor in the left breast, with clinical involvement of axillary nodes (cT3N3). Pathological evaluation of a core needle biopsy revealed the presence of HR-/HER2+ invasive ductal carcinoma (IDC). The patient received neoadjuvant CT with 12 cycles of weekly paclitaxel plus trastuzumab followed by 4 cycles of cyclophosphamide, epirubicin, and fluorouracil (FEC). She then underwent left mastectomy plus axillary node dissection (AND). Pathological study of the surgical specimen showed scattered foci of ductal carcinoma *in situ*, with no residual disease on axillary nodes (ypTisN0). The patient also underwent radiotherapy (RT) to the chest wall and supra-clavicular fossa. In addition, she received trastuzumab to complete 1 year.

**Figure 1:**
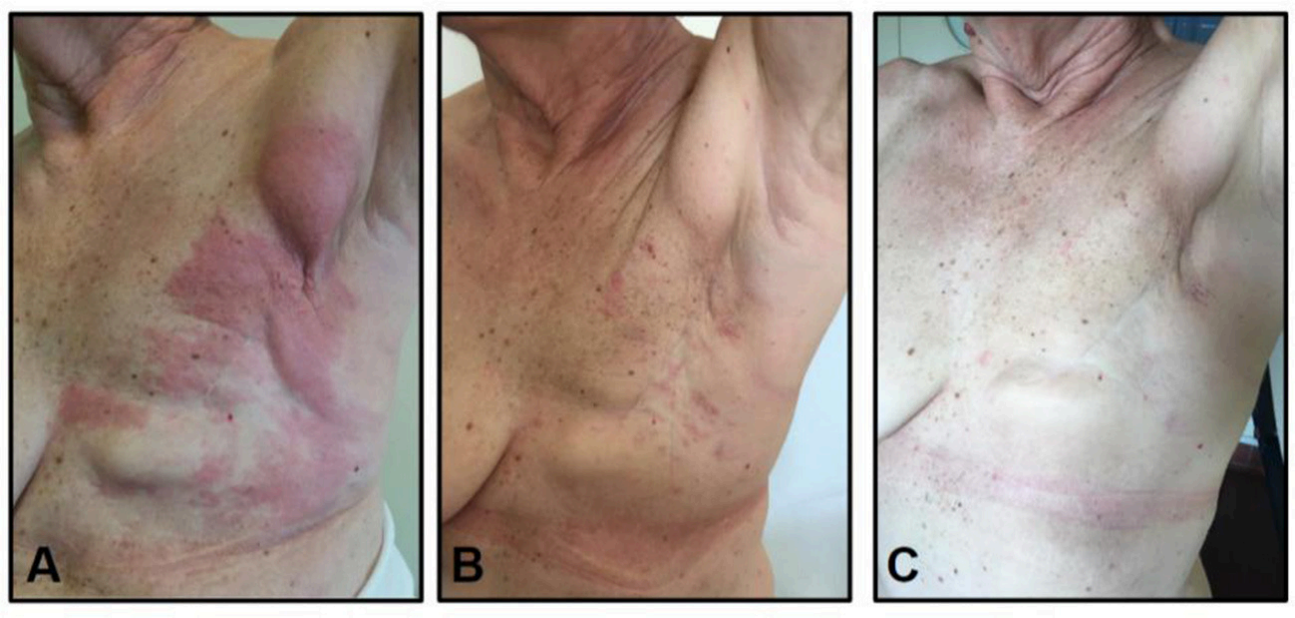
Evolution of Case 1-cutaneous metastasis: **(A)** before TDM1 initiation, **(B)** after 3 courses of TDM1, **(C)** after 45 courses of TDM1.

Unfortunately, while being treated with trastuzumab, a red wide cutaneous rash appeared on her left chest wall. A biopsy of such lesions confirmed the presence of HER2+ skin recurrence. A restaging CT scan did not show any other sign of distant metastasis. TDM1 was therefore initiated. Over the course of 4 weeks, the rash completely resolved. It has been 45 cycles of TDM1 and the patient is still disease-free (Figure 1).

### Patient 2

A 39 years old pre-menopausal woman came to our attention with a locally advanced BC. The pathological evaluation revealed the presence of HR-/HER2+ IDC (cT3N2). The patient initially received 3 cycles of neoadjuvant FEC followed by docetaxel concurrent with trastuzumab; docetaxel was discontinued due to anaphylactic reaction. She then underwent left mastectomy plus AND. The pathological study of the surgical specimen reported the presence of scattered foci of residual IDC in the breast and the presence of metastasis in four axillary nodes (ypT1micN2).

After surgery, 1 year of trastuzumab treatment was completed. The patient also underwent RT to the chest wall and sovra-clavicular fossa. After 12 months from the end of adjuvant trastuzumab, the patient experienced isolated skin relapse. In particular, she presented with itchy erythematous skin lesions on her left chest wall. Since a restaging PET did not reported any sign of distant metastatic disease, a wide cutaneous surgical excision was performed. Pathological study of the surgical specimen confirmed the presence of HR-/HER2+ skin recurrence.

The patient was then offered systemic therapy with CT plus an anti HER2 agent. However, she refused treatment. The patient was therefore strictly followed-up.

**Figure 2:**
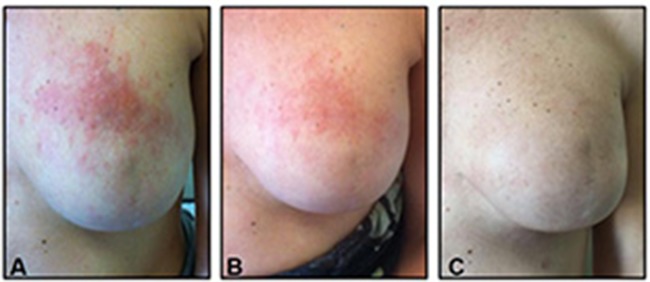
Evolution of Case 2-cutaneous metastasis: **(A)** before TDM1 initiation, **(B)** after 4 courses of TDM1, **(C)** 9 months after TDM1 discontinuation.

However, after a disease-free interval (DFI) of 4 months, the patient experienced a second skin relapse, with a wide erythematous rash appearing on her trunk. Systemic therapy with trastuzumab plus vinorelbine was therefore administered. Unfortunately, 5 months later, the patient experienced a cutaneous disease progression, consisting in an increasing in size of pre-existing skin lesions and appearance of new skin lesions on the antero-lateral abdominal wall. TDM1 was then initiated. Two months later, a complete response was achieved. After 17 cycles of TDM1, the patient decided to stop treatment. After 9 months since TDM1 had been discontinued the patient was still disease free (Figure 2).

## Discussion

These two cases show outlier responses of skin metastases to the anti-HER2 agent TDM1 following progression during or after trastuzumab-based therapy.

The introduction of anti-HER2 treatments has dramatically changed the outcome of HER2+ BC patients. However, still some patients relapse despite adequate treatment for early disease and still some patients with advanced disease experience poor outcome.

In particular, patients experiencing rapid progression on or after adjuvant trastuzumab or first-line anti-HER2 treatment in metastatic disease represent a challenging clinical scenario. Moreover, as previously discussed, cutaneous metastases represent a clinical challenge.

In all cases described herein, patients developed cutaneous lesions in the form of erythematous rash. This appears to be in agreement with a recent retrospective analysis that suggested that the patterns of skin involvement might reflect BC subtype, with erythematous plaques predominantly shown in HER2+ and TN BC, and skin ulceration or mass-forming lesions more frequently observed in HR+ BC (20).

All the cases presented here experienced relapse or progression during trastuzumab.

T-DM1 is a standard treatment in patients progressing on or early after trastuzumab, with median progression-free survival (PFS) from the Emilia pivotal trial of 9.6 months (16).

The cases presented here showed complete responses to T-DM1 which are maintained after 135 and 87 weeks, respectively, from T-DM1 starting. The first patient is still on treatment, while the second patient decided to interrupt it but still maintains the excellent response.

These outlier responses led us to hypothesize some of the mechanisms that might be responsible of these observations.

It is now well-accepted that the action of trastuzumab is, at least in part, exerted through an immune mechanism known as ADCC, consisting in tumor cell killing by immune effectors, especially macrophages and natural killer cells (21).

It is therefore not surprising if several authors reported hampered ADCC as a crucial mechanism of resistance to trastuzumab (22, 23).

Indeed, in a pioneering study, Gennari et al. suggested that ADCC activity of peripheral blood leukocytes was the only statistically significant difference between trastuzumab-responding and non-responding patients (23).

In this context, since natural killer cells play a major role in the recognition and subsequent lysis of trastuzumab-coated-her2+ tumor cells, it has been suggested that the efficiency of NK system might actually be considered a hallmark of responsiveness to trastuzumab (beano 2008). In addition, Trastuzumab-induced immune activity relies also on the proper function of the antigen presentation process (24).

Interestingly, it has also been reported that HER2+ breast tumors enriched for immune function genes showed increased relapse-free survival (RFS) after adjuvant trastuzumab as compared to tumors not exhibiting these immune features (25).

As recently described, along with brain, eye, testes, and placenta, immune privilege may take place also in the cutaneous tissue, where several immunosuppressive phenomena take place. Among others, the most significant involved mechanisms are (i) the low or absent expression of MHC I-II molecules, involved in the antigen presentation process; (ii) the inhibition of NK activity, crucial effectors of the cytotoxic innate immune activity; (iii) the upregulation of immune-checkpoints such as PD-L1, that turn down lymphocyte T activity (26–28).

It is therefore conceivable that such mitigation of the cytotoxic immune activity physiologically occurring in the skin could contribute to mediate resistance to trastuzumab.

**Figure 3:**
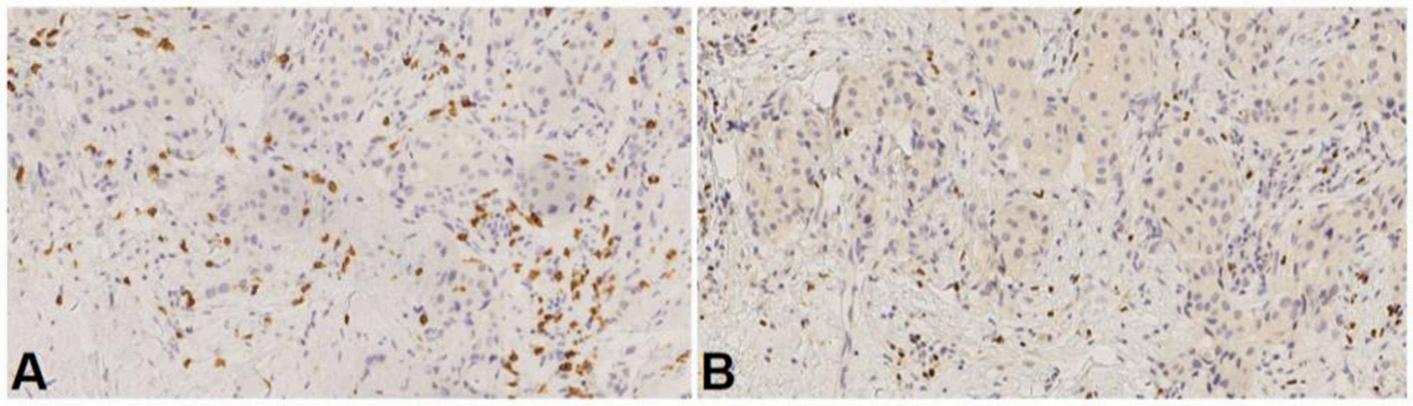
CD8/FOXP3 ratio from case 2 skin lesion. **(A)** CD8 antibody staining, **(B)** FOXP3 antibody staining. Ratio = 1,93.

Interestingly, we recently reported an analysis of immune microenvironment across BC metastases, showing that skin lesions exhibited higher FOXP3 levels and lower CD8/FOXP3 ratios as compared to other metastatic sites, especially in the HER2+ subpopulations, thus further suggesting that cutaneous tumor microenvironment may be unbalanced toward a more immunosuppressive phenotype (29). As an illustration, we show here the CD8/FOXP3 ratio in the skin lesion sample from case 2 patient. We observed CD8/FOXP3 ratio below 3, the cut-off that was used to discriminate high vs. low CD8/FOXP3 ratio in our previous publication (29) (Figure 3).

In this context, given its dual nature, the actual antitumor effect of TDM1 might depend in a less extent on its trastuzumab part, and conversely be predominantly due to its DM1 component. Accordingly, traditional resistance mechanisms to trastuzumab may not be sufficient to hamper TDM1 action. Therefore, if an immunosuppressive milieu might be crucial in selecting trastuzumab-resistant tumor cells, it may not be enough to contrast the antitumor effect of TDM1.

In addition, available data suggest that TDM1 may have immunostimulating properties. Indeed, TDM1 has recently been shown to potently enhance the immune system against tumor cells, thanks to the combined effect of trastuzumab and emtasine (30). In a mouse model of trastuzumab-resistant HER2+ BC, T-DM1 was effective and was associated with an increase in immune effector cells infiltrating the tumor. The most striking results in terms of efficacy and immune activation were observed with T-DM1 combined with CTLA-4/PD-1 blocking antibodies.

In conclusion, the cases presented here confirm the efficacy of T-DM1 with current indication and describe exceptional responses of isolated skin metastases. An intriguing suggested hypothesis might be that the immunosuppressive environment that might characterize some skin metastases could be detrimental for trastuzumab, preventing it from eliciting ADCC, but not for TDM1, that relies also on the additional action of DM1. This hypothesis deserves further research.

## Ethical considerations

All patients gave written informed consent for participation and publication of this case report in accordance with the Declaration of Helsinki.

## Author contributions

All authors (TG, FM, CG, VT, SM, LE, IP, MD, VG) contributed to: data collection, critical revision of the paper, and final approval of the version to be published. TG, FM, CG, MD, and VG conceived the work and wrote the paper.

### Conflict of interest statement

The authors declare that the research was conducted in the absence of any commercial or financial relationships that could be construed as a potential conflict of interest.

## Abbreviations

BC: breast cancer
LRR: locoregional recurrence
CT: chemotherapy
ET: endocrine therapy
DFS: disease-free survival
OS: overall survival
HR: hormone receptor
MBC: metastatic breast cancer
T-DM1: trastuzumab emtansine
HER2+: Human epidermal growth factor receptor-2 positive
IHC: immunohistochemistry
IDC: invasive ductal carcinoma
RT: radiotherapy
DFI: disease-free interval
PFS: progression-free survival
NK: natural killer cell
CTLA-4/PD-1: cytotoxic T-lymphocyte–associated antigen 4/ programmed death 1
FOXP3: Forkhead box protein 3
MHC: major histocompatibility complex
RFS: relapse-free survival.
